# The Oxygen Transport Triad in High-Altitude Pulmonary Edema: A Perspective from the High Andes

**DOI:** 10.3390/ijerph18147619

**Published:** 2021-07-17

**Authors:** Gustavo Zubieta-Calleja, Natalia Zubieta-DeUrioste

**Affiliations:** High Altitude Pulmonary and Pathology Institute (HAPPI-IPPA), Av. Copacabana Prolongacion #55, La Paz 2826, Bolivia; n_zubieta@yahoo.com

**Keywords:** high altitude, chronic hypobaric hypoxia, physiologic adaptation, travel to high-altitude cities, high-altitude physiology, pulmonary hypertension, cardiorespiratory test, acute sea-level sickness, high-altitude gas expansion, lung disease, hemoglobin, tolerance to hypoxia, oxygen content

## Abstract

Acute high-altitude illnesses are of great concern for physicians and people traveling to high altitude. Our recent article “Acute Mountain Sickness, High-Altitude Pulmonary Edema and High-Altitude Cerebral Edema, a View from the High Andes” was questioned by some sea-level high-altitude experts. As a result of this, we answer some observations and further explain our opinion on these diseases. High-Altitude Pulmonary Edema (HAPE) can be better understood through the Oxygen Transport Triad, which involves the pneumo-dynamic pump (ventilation), the hemo-dynamic pump (heart and circulation), and hemoglobin. The two pumps are the first physiologic response upon initial exposure to hypobaric hypoxia. Hemoglobin is the balancing energy-saving time-evolving equilibrating factor. The acid-base balance must be adequately interpreted using the high-altitude Van Slyke correction factors. Pulse-oximetry measurements during breath-holding at high altitude allow for the evaluation of high altitude diseases. The Tolerance to Hypoxia Formula shows that, paradoxically, the higher the altitude, the more tolerance to hypoxia. In order to survive, all organisms adapt physiologically and optimally to the high-altitude environment, and there cannot be any “loss of adaptation”. A favorable evolution in HAPE and pulmonary hypertension can result from the oxygen treatment along with other measures.

## 1. Introduction

Concerning our article entitled “Acute Mountain Sickness, High-Altitude Pulmonary Edema, and High-Altitude Cerebral Edema: A View from the High Andes” [1], we now include further comments with clarifications. This opinion article results from observations raised by a delimited group of “sea-level high-altitude experts”. The questions asked covered topics such as: the use of brackets instead of the multiplier x in the CaO_2_ formula, not mentioning oxygen diffusion to mitochondria, and arterial pH returning to normal at high altitude. Also, their disagreement with the use of the terms High-Altitude Flatus Expulsion (HAFE) and High-Altitude Gas Expansion (HAGE), the precedence of High-Altitude Pulmonary Edema (HAPE) and High-Altitude Cerebral Edema (HACE) by Acute Mountain Sickness (AMS), the non-mention in our 100% successful treatments of their use of multiple drugs like glucocorticoids, nifedipine or other calcium blockers, sildenafil or PDE5 inhibitors, our lower incidence of COVID at high altitude, and some other non-transcendental observations. Furthermore, the following statement was written: “the author clearly overlooked that there is some definite vital risk of permanent living at high altitude, as between 5 to 15% of the high Andean population suffer from Monge’s disease, a ‘disadaptation’ to high-altitude conditions”.

This article answers those questions and further expands our points of view based on our successful treatments as high-altitude physicians with 50 years of experience at our High Altitude and Pulmonary Pathology Institute (HAPPI-IPPA), located in La Paz, Bolivia, 3500 m.

Many authors use the term “acclimatization” to high altitude. We believe that this term’s use should be referred only to climate changes, not hypoxic altitude changes. Climate changes involve abiotic qualities of ecosystems, e.g., temperature, humidity, and CO_2_ concentration [2]. Those are dynamic variables and depend on the weather. Whereas a fixed Partial Inspired Oxygen Tension (PIO_2_), due to a lower barometric pressure at high altitude, significantly impacts living organisms as it involves oxygen—the fundamental element of the respiratory chain, a variable not included in climate change. The stimulus of a lower PIO_2_ induces “physiologic adaptation”. According to Dictionary.com, the word adaptation is defined as: “A change in structure, function, or behavior by which a species or individual improves its chance of survival in a specific (new) environment”. We are aware that geneticists have previously defined “adaptation” solely as a process associated with genetic modifications. However, besides ourselves, several authors likewise use the concept of physiologic adaptation in reference to high altitude [3,4]. We believe that adaptation should be defined based on two types: Genetic and Physiologic: “Genetic adaptation” as the process of changes of form or behavior brought about through natural selection and genetic modification (millions or thousands of years of molecular genetic evolution); and “Physiologic adaptation” as the process of adjusting to environmental circumstances through epigenetic expressions and physiological immediate or delayed responses, making in situ survival possible (within one lifetime). The epigenetic modifications (physiological adaptation) that change DNA expression at high altitude are well described [3]. “Biological adaptation” is a term used to refer to seals’ breath-holding for over 1 h during deep-diving [5].

The establishment of the concept of adaptation as solely genetic has given rise to biased misinterpretations. One of them is that prolonged high-altitude hypoxia exposure leads to progressive deterioration, referred to as maladaptation or disadaptation [6,7]. Nevertheless, we disagree with this concept since over 200 million people live and work above 2000 m of high altitude with no life threat developed over time [8,9]. High-altitude-generated diseases are not adequately understood thus far. This happens due to a poor understanding of the body’s plasticity (or ability to change) and the physics laws applied to biology in a chronic hypoxia environment.

Analogous is the questionable belief that Chronic Mountain Sickness (CMS), or Monge’s Disease, at high altitude is due to a “Loss of Adaptation” [10,11,12,13]. This idea has led to a desperate and complicated search for genetic “malformations” in those patients [13,14]. However, their diseases are frequently related to altered cardiovascular function, also found at sea level [15]. It is more appropriate to refer to individual predisposition, “dysfunctional organs”, and underlying “silent” disease that can occur at high altitude, just like at sea level. For example, Type 1 Diabetes results from a dysfunctional pancreas. It is not a “loss of adaptation” at sea level. Chronic Mountain Sickness (CMS) is not an isolated disease but multiple diseases in the chronic hypoxic environment [16]. We think it would be more accurately denominated as “Chronic Sicknesses in the Mountain” (CSM). At our institute, we use the term “Poly-Erythrocythemia” (PEH), where Poly = many, Erythrocyt = Red Blood Cells, and hemia = in blood, to describe these alterations [16]. CMS, or better yet, PEH, should instead be addressed as a syndrome derived from the presence of one or several dysfunctions in the lungs, heart, kidneys, carotid bodies, central hypoventilation, hemoglobin alterations, and/or perhaps others in the chronic hypoxia environment presenting itself differently than at sea level. But it cannot be referred to as a “Loss of Adaptation” [16,17]. In biology, aging is an irreversible forward process, being impossible to grow younger. Analogously, in physics, time is irreversible, except in theoretical estimations. Similarly, the late Prof. Dr. Gustavo Zubieta-Castillo (1926–2015) proposed that adaptation is an ongoing dynamic vital process. More on this can be found under the Adaptation Formula section of this paper. Further insight on CMS will be explained in another article on the subject, currently under development.

Sea-level physicians and scientists occasionally visit high altitude for short periods of time to perform some studies and produce interesting results. However, to gain true expertise in the extensive field of high-altitude medicine and physiology, it is most practical to live and work permanently in the chronic hypobaric hypoxia environment. The adjacent cities of La Paz and El Alto are located between 3100 and 4100 m of altitude in Bolivia, in the heart of South America. They are the highest cities in the world with over 2 million inhabitants (https://www.visualcapitalist.com/the-50-highest-cities-in-the-world/ (accessed on 12 July 2021). We denominated La Paz as the “Capital of Hypoxia” and the “Shrine of Hypoxia Scientists” since the 1st Chronic Hypoxia Symposium carried out in 2005 (https://zuniv.net/symposium/ (accessed on 12 July 2021). We organize these symposiums every two years in La Paz, Bolivia, but the fourth was carried out in New Dehli, India. During the 7th Chronic Hypoxia Symposium, Prof. Pavel Beloshitsky, previous Head of the Elbrus Medico–Biological Scientific Centre in Terskol, Ukraine, also supported this denomination (https://zuniv.net/symposium7/Abstracts7CHS.pdf (accessed on 12 July 2021).

## 2. High-Altitude Physiology

In our previous article [1], we review high-altitude physiology based on our perspective on what is truly useful. We now further expand some essential aspects. Breathing physiology at high altitude differs from that at sea level. The arterial oxygen saturation (SaO_2_) in pulse-oximetry (SPO_2_) fluctuates with the change of breathing patterns, and a deep breath increases alveolar oxygen tension [18]. This is only evident at high altitude as the PaO_2_ is located in the steep portion of the hemoglobin oxygen dissociation curve. At sea level, since the oxygen pressures are high and the oxygen dissociation curve is in the top plateau, those changes cannot be observed. In a high-oxygen pressure environment as at sea level (PIO_2_ = 150 mmHg), a slight change in arterial partial pressure of oxygen (PaO_2_) does not change saturation (SpO_2_). This is one fundamental factor that confuses sea-level physiologists concerning high altitude.

## 3. The Oxygen Transport Triad

It is helpful to explain the Oxygen Transport Triad. What we define as the Oxygen Transport Triad has three components: the pneumo-dynamic pump (an air vacuum pump), the hemo-dynamic pump (a blood compression pump), and hemoglobin (the iron oxygen porter molecule in the blood). In order for the reader to interpret the role of the pneumo-dynamic (respiratory) and hemo-dynamic (cardio-vascular) pumps observed in our laboratory at 3500 m above sea level in La Paz, Bolivia, we developed, 21 years ago, a study measuring cardio-respiratory parameters under four conditions: the Resting Positional Change and Exercise Test (RPCET). This data was presented in a hypoxia conference organized by the Bogomoletz Physiology Institute in Ukraine in 2000.

**Figure 1:**
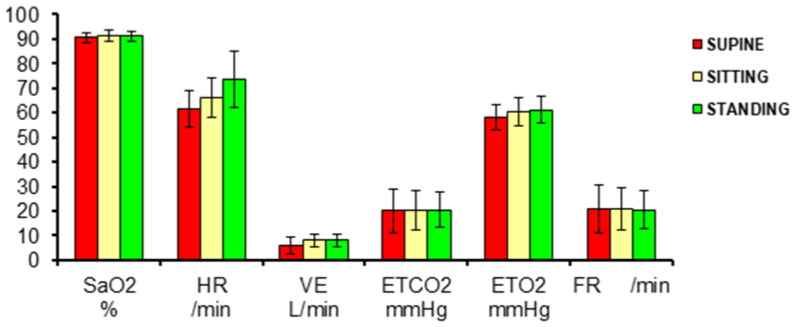
Ventilation and pulse-oximetry studies in the three resting positions: (A) supine (red), (B) sitting (yellow), (C) standing (green) in 15 healthy high-altitude men at 3500 m of altitude. SaO_2_ = arterial oxygen saturation from pulse-oximeter, HR = heart rate, VE = ventilation, Body Temperature, Pressure water vapor Saturated (BTPS), ETCO_2_ = end-tidal carbon dioxide, ETO_2_ = end-tidal oxygen, FR = respiratory frequency.

In the RPCET, the subject is first studied at rest under three conditions: (A) lying down, (B) sitting, (C) standing up, and then during a five-stage modified Bruce exercise protocol (Figure 1). The three resting positions were studied for 6 min each in 15 healthy native adult high-altitude male residents at 3500 m. Ventilation was measured by pneumotachograph, pulse and saturation by pulse-oximetry, and end-tidal O_2_ and CO_2_ from expired gas. The mean values and standard deviations found are presented.

**Figure 2:**
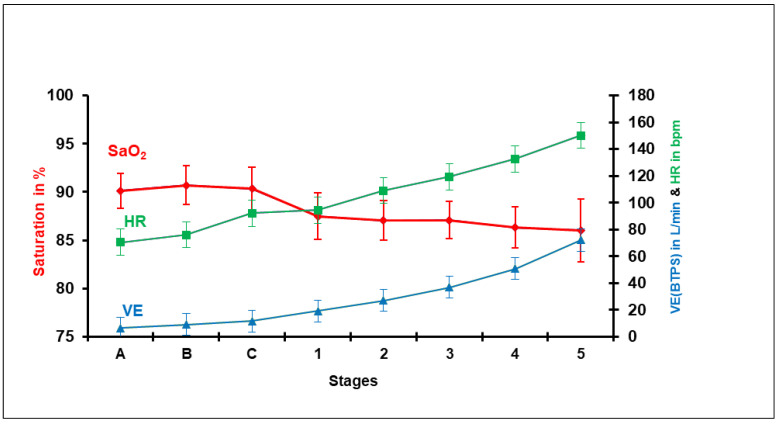
Mean values and standard deviation of oxygen saturation (SaO_2_) obtained by pulse-oximetry, heart rate (HR), and ventilation (VE) in Body Temperature, Pressure water vapor Saturated (BTPS) during the three resting stages, (A) supine, (B) sitting, and (C) standing, and then during the five stages of exercise using the Modified Bruce exercise protocol mph/gradient (0/0, 2/0, 2/5, 2/10, 3/12, 4/14) in 15 healthy subjects.

Comparative supine, sitting, and standing studies in sex and age differences at sea level show the heart-rate increase [19]. While lying down, the respiratory and cardiac muscles are active and constitute the basal metabolic rate (including the brain and other organs’ basal oxygen consumption). In the sitting and standing positions, the pneumo-dynamic pump and the hemo-dynamic pump gradually increase ventilation and heart rate, respectively, to maintain the oxygen saturation related to the arterial blood oxygen content. The change of positions requires the use of additional orthostatic muscles and, hence, greater oxygen consumption. While sitting down, some muscles in the back are involved in maintaining the thorax up, whereas standing up, the orthostatic muscles of the legs and thighs add up. In all three positions, the brain and all other organs seem to maintain a similar oxygen consumption. The oxygen consumption is adequately compensated for the gradually increasing metabolic requirement in the three resting positions keeping SpO_2_ within normal limits. During exercise at different incremental work levels at 3500 m, the SpO_2_ is steadily reduced due to increased oxygen consumption (Figure 2). It is interesting to point out that in space flight humans do not use the orthostatic muscles and, consequently, the hematocrit reduces [20], and it is a crucial BioSpaceForming factor [21,22].

**Figure 3:**
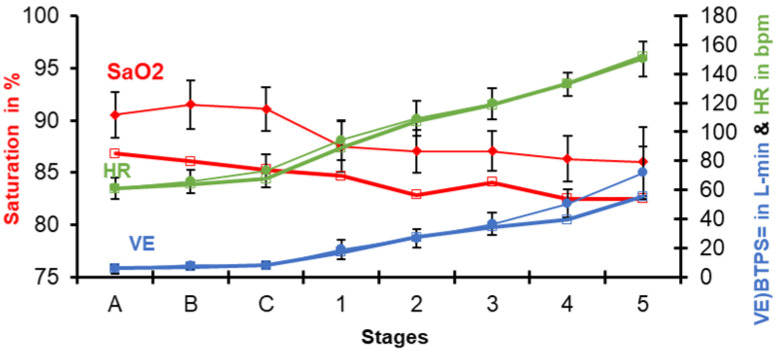
Same procedure as in Figure 2, this time comparing the previous mean values of 15 healthy subjects with those of a patient with Poly-ErythroCythemia (PEH) (in thick lines) at 3500 m.

When the pumps are not fully effective (dysfunctional), as in cardio-pulmonary disease, the saturation drops; however, in chronic hypoxia, the oxygen content is maintained by the physiological adaptation mechanism of increasing the number of red blood cells (hemoglobin and hematocrit). This is fundamental in the chronic hypobaric hypoxic environment at high altitude. By comparing the 15 healthy Andean men with an average Hematocrit (Ht) = 50% with one native adult male patient with poly-erythrocythemia (PEH) (Ht = 72%), it can be appreciated that the latter has a lower SpO_2_ at rest due to some cardiac or pulmonary disease. The hematocrit (the third oxygen transport factor) rises to compensate for the cardiopulmonary deficiencies. The two pumps work similarly to those of the normal hematocrit residents, but the SpO_2_ is decreased. However, the test follows a similar behavior with a progressive reduction of SpO_2_ (Figure 3).

These studies are valuable for the diagnosis of cardio-pulmonary disease at high altitude. They help understand how CMS, or rather poly-erythrocythemia (PEH), is erroneously considered a single illness, actually it being multiple diseases that try to compensate cardio or respiratory inefficiencies (organ dysfunction) by increasing the red blood cells [16]. However, regardless of the elevated hematocrit, exercise is carried out and achieved as well as normal residents at 3500 m. This was observed by several authors [23,24].

**Figure 4:**
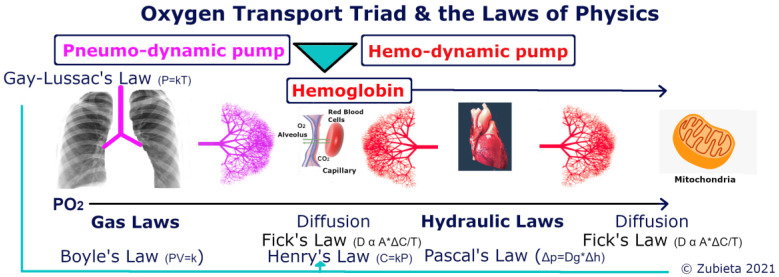
The Oxygen Transport Triad involves: the pneumo-dynamic pump, hemo-dynamic pump and Hemoglobin. The laws of physics applied to biology in each section are shown. It is important to note that Henry’s Law is effective in all sections: PIO_2_, PAO_2_, PaO_2,_ PtO_2_, and PmO_2_.

It is important to note that the first component of the Oxygen Transport Triad “moves” oxygen from the environment to the lungs with the vacuum produced in the pneumo-dynamic pump [25]. Oxygen is then transferred through the alveolar-capillary membrane by “diffusion and only by diffusion”, quoting the great August Krogh [26]. Oxygen is subsequently captured by hemoglobin. After that, the hemo-dynamic pump moves the hemoglobin oxygen-filled blood through a compression mechanism (the heart) to the tissue capillaries. The diffusion process then repeats itself at the cellular level to reach the mitochondria. At any altitude, biology follows the laws of physics within the Oxygen Transport Triad (Figure 4). As air enters the upper airway tract from an environmental PIO_2_, it increases volume from Ambient Temperature Pressure Standard (ATPS) to Body Temperature Pressure water vapor Saturated (BTPS) due to temperature and humidity change (Gay-Lussac’s Law and Henry’s Law). As air enters the lung, where there is a negative vacuum pressure, it expands (Boyle’s Law), where it has an Alveolar Partial Pressure of Oxygen (PAO_2_). Oxygen is then transferred through the alveolar-capillary surface area membrane to hemoglobin due to a positive pressure concentration gradient through diffusion (Fick’s Law). Once in the blood, there is an Arterial Partial Pressure of Oxygen, (PaO_2)_ (Henry’s Law), and a CaO_2_ with oxyhemoglobin and plasma-dissolved oxygen. The blood is then transported from the lung capillaries to the tissues, tissue partial pressure of oxygen (PtO_2_) through hydraulic pressure changes (Pascal’s Law). The oxygen is finally transported to the mitochondria, that should be denominated as mitochondrial partial pressure of oxygen (PmO_2_) through diffusion (Fick’s Law again).

## 4. The Arterial Oxygen Content (CaO_2_) at High Altitude

The Arterial Oxygen Content in the blood can be calculated from:

CaO_2_ = (SaO_2_ × 1.34 × Hb) + (0.003 × PaO_2_)

(O_2_ in hemoglobin) + (Dissolved O_2_ in plasma).

In the original article, we used: CaO_2_ = SaO_2_ × 1.34 × Hb + 0.003 (PaO_2_), which is mathematically correct.

It is important to note here that hemoglobin, the miracle molecule, transports practically all the arterial oxygen content in the blood. Interestingly, at sea level, the CaO_2_ = 20 Vol% O_2,_ and hemoglobin transports 98.45% of the oxygen. Breathing ambient air at sea level (760 mmHg), the dissolved oxygen portion of the CaO_2_ constitutes only 1.55% of the total oxygen content. Whereas at high altitude 3500 m (495 mmHg), the CaO_2_ = 20.50 Vol% O_2_ (more elevated than at sea level), and hemoglobin transports 99.13% of the oxygen whereas dissolved oxygen only 0.87% (half of that at sea level), Table 1. Hence the importance of hemoglobin’s elevation at high altitude for efficient oxygen transport [27].

## 5. The Ferromagnetism of Iron in Hemoglobin and the Paramagnetic Property of Oxygen

It is important to note that oxygen, the fundamental element of respiration and hence life, is linked to the most stable element in the universe: iron. It is linked to globin forming the well-studied complex hemoglobin molecule. Deoxygenated hemoglobin is paramagnetic, whereas oxygenated hemoglobin is diamagnetic [28]. Oxygen has paramagnetic properties, being captured by electromagnets in its liquid form (for visualization). Some of the most efficient fast oxygen sensors are based on these paramagnetic characteristics. At high altitude there is higher hemoglobin as a compensating hypobaric environmental oxygen pressure [27]. With a more elevated hematocrit, it is possible that the paramagnetic properties of deoxygenated hemoglobin increase, thereby facilitating the capture of oxygen. This needs to be studied, as it would help explain an additional reason of the important elevation of hemoglobin at high altitude.

## 6. The High-Altitude Adaptation Formula

Concerning the High-Altitude Adaptation Formula that we created:

Adaptation = time/altitude Δ.

It is a fundamental concept of adaptation to high altitude: as one travels higher (greater altitude change (Δ)), more time is needed to achieve an adequate physiological adaptation [27]. It refers to the hemoglobin rise where “Full hematological adaptation to high altitude is achieved when the increase of red blood cells reaches a plateau and stops”. Hemoglobin increases logarithmically increasing over time. In other words, the organism wisely decides to increase the number of red blood cells to optimal levels, thereby reducing the extra load and energy-consuming hyperventilation (pneumo-dynamic pump) and tachycardia (hemo-dynamic pump). We have termed this “the most energy-efficient adaptation mechanism to high altitude”, even in Chronic Mountain Sickness [29]. Gustavo Zubieta-Castillo Sr (1926–2015) wrote a transcendental concept from where this adaptation formula derives: “The organic systems of human beings and all other species tend to adapt to any environmental change and circumstance within an optimal period of time, and never tend towards regression which would inevitably lead to death” [16]. Regression would be equivalent to “Loss of Adaptation”, which we believe is a wrong interpretation, often still used.

## 7. Acid-Base Balance at High Altitude

Another important aspect related to physiologic adaptation is the acid-base status of blood. It plays an essential role at high altitude but, unfortunately, it is poorly understood. Some studies have shown that pH remains alkalotic in visitors from sea level to 5260 m but not in high-altitude inhabitants [30]. Furthermore, there is the erroneous belief among some sea-level physiologists that all high-altitude residents live in an altered acid-base status characterized as Respiratory Alkalosis, based solely on the decrease of the arterial partial pressure of carbon dioxide (PaO_2_). This is a questionable concept as all high-altitude inhabitants need to adapt and live in a perfectly balanced acid-base status to sustain life. Thousands of metabolic and enzymatic reactions in cells require a perfectly balanced pH = 7.4. The body achieves this by decreasing blood bicarbonate (HCO_3_) due to the decrease of PaCO_2_ at high altitude [31]. We established the Van Slyke formula’s correction factors at high altitude to adequately interpret acid base following the Ole Siggaard-Andersen sea-level concepts [31]. These concepts were later confirmed by other authors [32,33]. This is why we wrote an article questioning how over 200 million high-altitude inhabitants on planet Earth could live permanently in an altered acid-base status [8]. We, likewise, questioned the use of sea-level interpretation of acid-base status on arterial blood gas samples taken at 8400 m near the summit of Mt. Everest [34]. In that article, the Base Excess (BE) for the four Mount Everest climbers was calculated as an average to be −6.9 mM, using sea-level parameters. This is an abnormal acid-base status. However, using our correction factors, it was possible to demonstrate a normal acid-base status in the successful climbers of the world’s highest peak [35]. THID (Titratable Hydrogen Ion Difference = -BE) was calculated to be 0.7 mM, close to a perfect acid-base balance of “0”. Such values are excellent for optimal cellular metabolism and enzymatic reactions. To the best of our knowledge, no other possible up-to-date explanation exists to explain a successful climb to the summit of Mt. Everest. Consequently, acid-base balance plays a fundamental role in acute high-altitude diseases, but it has to be adequately interpreted following our correction factors [31].

## 8. Variations of Pulse-Oximetry at High Altitude

One of the fundamental measurements in high-altitude medicine is pulse-oximetry. Since SpO_2_ at high altitude is located in the sloped part of the hemoglobin oxygen dissociation curve, breathing variations can increase pulse-oximetry changes. This is why it is important to show those variations with a breath-holding test we developed [36]. It is essential to consider these variations when evaluating a person who is suffering from high-altitude illness. After a deep inspiration followed by breath holding, as long as possible, in the city of La Paz, at 3500 m, the pulse-oximetry (SpO_2_) increases from a normal 90% up to 98% a sea-level value. This is achieved because on deep inspiration the physiological dead space (VDphys) is reduced by dilution. The VDphys/Tidal Volume ratio is increased, thereby increasing the PaO_2_ and SpO_2_.

## 9. The Expansion of Gases at High Altitude

Regarding the expansion of gases at high altitude: Joseph Hamel, a scientist from Geneva, was the first to describe High-Altitude Flatus Expulsion (HAFE). HAFE was also mentioned by the notable Paul Auerbach, the respected past president of Wilderness and Environmental Medicine [37]. We propose a new terminology: HAGE (High-Altitude Gas Expansion), based on the very fundamental Boyle’s law of gases and pressures. At high altitude, all physics laws stand solid, playing an essential role in biological adaptation. We calculated the theoretical expansion of gases in the abdomen, which is very important. In the city of La Paz, at 3600 m (11,811 ft), according to Boyle’s Law of Gases: due to the lower barometric pressure, maintaining the same temperature (i.e., body temperature = 37 °C), P1 × V1 = P2 × V2 results in 760 mmHg × 1 Lt (gas)/495 mmHg = 1.5. This means that the volume of gases can expand 50% more than at sea level.

The ideal gas law PV = nRT stands for any altitude [38]. Metabolic gas output from GI digestive chemical reactions should be the same at any altitude. However, due to the lower pressure, the flatulence gas volume should grow and be larger at high altitude, if the same volume were to be permanently produced. 

The high-altitude flatulence was also mentioned in an article on the High Andes, *National Geographic* magazine April 1987, Volume 171 No. 4, where Loren McIntyre interviewed Gustavo Sr. and Jr., referring to it as “breaking the wind”.

Furthermore, it is essential to note that gases’ distension upon arriving at high altitude is not a transient phenomenon. The ideal gas law PV = nRT stands for any altitude. Metabolic gas output from GI digestive chemical reactions has to be the same at any altitude. However, due to the lower barometric pressure, the flatulence gas volume grows and is more prominent at high altitude permanently. Some believe that “once at a stable altitude, the differential pressure between the atmosphere and GI tract disappears,” as mentioned in one observation raised by a delimited group of “sea-level high-altitude experts” to our original paper [1] but this is incorrect as it only applies to non-organic things such as bottles or balloons. Biology is linked to the pressure laws of physics at any altitude. This concept could help explain what Frisancho has described as the Dolicomegacolon (a loop of intestine twists around itself and the mesentery that supports it, resulting in a bowel obstruction—often a medical-surgical emergency) quite common in high-altitude natives residing at 3800 m or above [39,40].

## 10. The Tolerance to Hypoxia Formula

Acute exposure to hypobaric hypoxia is different than acute exposure to normobaric hypoxia. This has to do with the denominator of our formula:

Tolerance to hypoxia = Hb/PaCO_2_.

Several studies have found differences between hypobaric (high-altitude) hypoxia and normobaric (sea-level) hypoxia artificially produced through a reduced Inspired Fraction of Oxygen (FIO_2_) [41,42]. There are many confounding factors, but everyone seems to forget the role of a reduced PaCO_2_ at high altitude. This is a critical factor, as a lower PaCO_2_ gives rise to higher tolerance to hypoxia [43,44]. We have compared exercise studies in the city of La Paz, breathing ambient air and then simulating the Chacaltaya altitude of 5300 m in our Hyperoxic/Hypoxic Adaptation Chamber. We then went to Chacaltaya, where our Glass Pyramid Laboratory (the highest in the world, easily accessible by road 2 h from our institute located in La Paz, Bolivia). We repeated the exercise studies in the same subjects, and we showed differences with the normobaric hypoxia at 3500 m in our lab in La Paz.

It is interesting to note that normobaric hypoxia is always referred to as a barometric pressure of 760 mmHg at sea level. However, we live in La Paz with a barometric pressure of 494 mmHg. This is normobaria for us. From our perspective, it is normoxia because we are born, develop, practice sports, study, get married, reproduce, and live to old age at high altitude. Consequently, someone who has never gone to sea level considers himself to be living in a “normal normoxic normobaric environment”. We consider going to sea level, an exposure to “hyperoxia” and “hyperbaria” and some of us can present some temporary difficulties adapting to sea level [20]. Those alterations could be termed Acute Sea Level Sickness (ASLS). The symptoms observed include sleepiness, edema in the lower limbs, hypotension, fatigue, and drowsiness. We have named the Godett positive edema in the lower limbs that appeared after going down to sea level as LAPE (Low-Altitude Peripheral Edema) [45]. More studies on this area are being performed.

## 11. The Oxygen Levels in the Summit of Mt. Everest and Fetuses

Babies in the maternal womb have a PaO_2_ of 30 mmHg similar to that found at the summit of Mt. Everest [34]. They live at the hypoxic levels of Mt. Everest. Furthermore, poly-erythrocythemia (CMS) patients during the Triple Hypoxia Syndrome [46] have a PaO_2_ of 30 mmHg or lower. They are living as though they were at the hypoxic levels of the summit of Mt. Everest. Every human being on planet Earth has lived at the hypoxic levels found on top of Mt. Everest in the womb. According to our Tolerance to Hypoxia Formula [43], a subject on the summit of Mt. Everest tolerates close to six times more hypoxia than a sea-level resident. The limiting factors for climbers to the summit of Mt. Everest include cold, fatigue, lack of sleep, and malnutrition [47]. As expected, all these factors bring forth a potentially temporary progressive physiological deterioration but, unfortunately, it is only attributed to hypoxia, an often blind and biased sea-level approach.

## 12. AMS Evolving to HAPE and/or HACE

**Figure 5:**
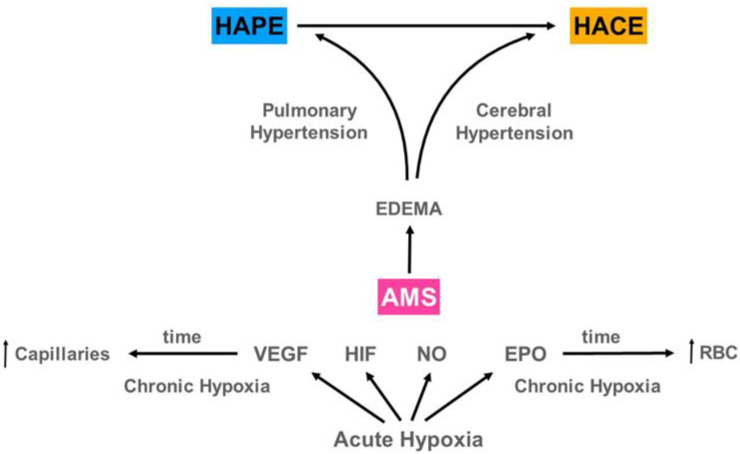
We postulate that AMS on exposure to high-altitude hypoxia can sometimes evolve to either High Altitude Pulmonary Edema (HAPE) and/or High Altitude Cerebral Edema (HACE). After recovery or if there is no altitude illness, the evolution towards an increase of hemoglobin, hematocrit, and red blood cells and the formation of more capillaries makes the organism of high-altitude residents resistant to several diseases and can even lead to extended longevity. VEGF = Vascular Endothelial Growth Factor, HIF = Hypoxia Inducing Factor, NO = Nitric Oxide, EPO = Erythropoietin, RBC = Red Blood Cells.

Acute Mountain Sickness (AMS) results from exposure to acute hypoxia when ascending to a higher altitude. AMS symptoms include sleeping disorders, uneasiness, dehydration from hyperventilation and diminished thirst sensation, loss of appetite, and fatigue upon physical activities. When it pertains to arriving at a city like La Paz (3500 m), perhaps it should be referred to as Acute Hypoxia Sickness (AHS). The term AMS refers to all-mountain (hypoxia) sickness on acute exposure. This would etymologically include High-Altitude Pulmonary Edema (HAPE) and High-Altitude Cerebral Edema (HACE) that also form part of the term “acute mountain sickness” but in a more severe or advanced form, in our criteria. Most HAPE and HACE patients manifest several of the AMS symptoms. HAPE can be preceded by AMS, although some would disagree. We consider that AMS is the first step, and it can be aggravated and evolve to the two more advanced forms and, possibly, lethal conditions: HAPE and HACE (Figure 5). Not everyone has a headache in AMS, so it is not reasonable to discard the diagnosis of AMS if the headache is not present in HAPE. However, headaches are present in several HAPE patients, along with shortness of breath, cyanosis, anorexia, and general malaise.

## 13. Treatment of HAPE and HACE at High Altitude

We are highly successful in our treatments of high-altitude diseases. We have saved all our high-altitude patients’ lives and saved travel insurance companies extraordinary expenses in the hundreds of thousands of dollars cost for airplane ambulances or millions of dollars for the loss of life. Rest is fundamental, yet we have never treated the patients solely with rest, although Marticorena and Hultgren wrote and published about the proven efficacy of rest in La Oroya [48,49]. Oxygen treatment is basic and extremely important. Oxygen is the essential physiological pulmonary circulation vasodilator that rapidly reverses hypoxic pulmonary vasoconstriction. Pulmonary artery pressure typically returns to normal levels within minutes [50]. We have had success following our methods. And the article in reference [1] is a paper explaining our experience. It has not been necessary in our treatments to use multiple drugs like glucocorticoids, nifedipine or other calcium blockers, sildenafil, or PDE5 inhibitors, which may be required in very particular cases. Treatment in these diseases is fundamentally based on the individual etiology found after performing a general check up. Glucocorticoids can be used in advanced cases of HACE.

## 14. Pulmonary Hypertension and a Dilated Right Ventricle in (HAPE)

**Figure 6:**
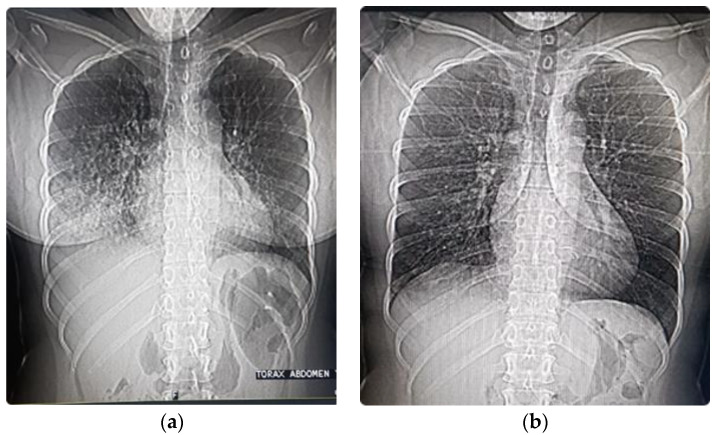
Thorax and abdominal X-rays performed during the CAT scan of a patient with HAPE at 3500 m. (**a**) Four days after arrival at high altitude. Note the distension of gases HAGE; (**b**) Two days later following the treatment described in our article [1].

In HAPE, the lungs present edema with a cotton image in chest X-rays and chest CAT scans (Figure 6a). The pulmonary edema reduces the gas exchange surface area and produces aggravating hypoxia. This functional limitation has some similarities with COVID pneumolysis (pneumo = lung, lysis = destruction), where there is progressive silent hypoxemia until there is extreme hypoxia and hypercapnia [51]. Whereas post-COVID patients can have pulmonary fibrosis due to pneumolysis [25], in HAPE, recovery post-edema leaves no sequelae following prompt resolution within a few days (Figure 6b).

HAPE presents with pulmonary hypertension that can be excessive in some people on exposure to hypoxia [52,53]. Furthermore, some individuals who previously presented HAPE were shown to have an excessive pulmonary vascular constriction not wholly responsive to oxygen administration [53], but nevertheless partially responsive and resulting in a favorable evolution.

The COVID-19 pandemic has made it evident that previously feared extreme hypoxia can happen at sea level and is called “Silent Hypoxemia” [51]. Several authors initially thought that there might be similarities between COVID-19 and HAPE. However, others criticized their point of view, affirming that there is no similarity [54,55]. Nevertheless, it is essential to point out that although they are different diseases, and we agree you cannot use the same medication [56], oxygen is one of the fundamental treatments in both. If tissue mitochondria receive enough oxygen, while immunity blocks further pneumolysis attack of COVID and reduces pulmonary hypertension (similar to what happens in HAPE), recovery is possible. Impaired gas exchange and hypoxemia in both pathologies are the underlying health issues that can be deadly. Although the etiology of these diseases is not the same, the pathophysiology of hypoxia is quite similar. As the gas exchange surface is gradually reduced with an evolving disease, the hypoxemia decreases linearly, as in COVID-19 [51]. In HAPE, there is the formation of edema that hinders the transport of oxygen from the alveoli to the capillaries [57,58]. This would be a diffusion alteration [59].

Similarly, in COVID, due to pneumolysis (lung destruction due to COVID) [25], there is superimposed inflammation that can result in ventilation –perfusion anomalies, diffusion alteration, and shunt [51,60]. Fatal pulmonary fibrosis can ensue [61]. In HAPE, after recovery in a couple of days, or perhaps a few days more, recovery is complete as there is no destruction of the alveoli. Whereas in COVID-19, the pneumolysis evolves gradually and can be deadly due to incremental hypoxia. If the patient’s immune system is able to overcome the disease, pulmonary fibrosis can result, limiting the exercise capacity post-COVID [62].

**Figure 7:**
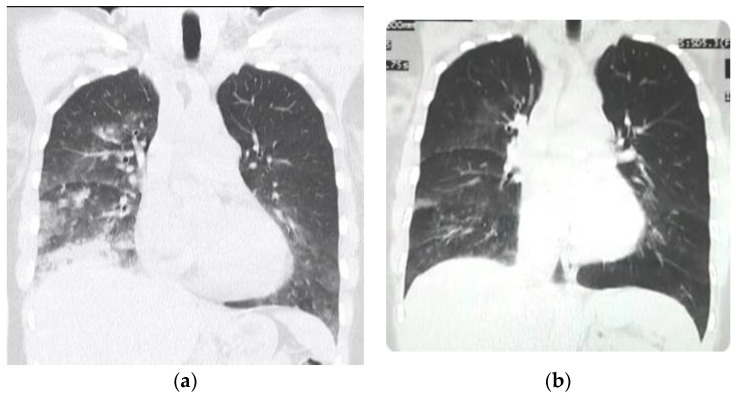
CAT scan of patient with HAPE. (**a**) Note the convex pulmonary arch due to pulmonary hypertension in the acute phase. The heart apex is likewise elevated due to dilatation of the right ventricle. (**b**) In the recovery stage of HAPE (two days later during treatment) a decrease of the pulmonary arch can be observed at closely similar CAT scan coronal cut level (the size of the heart, due to systole or diastole, may have variations).

The electrocardiogram in HAPE reports sinus tachycardia (increased activity of the hemo-dynamic pump) and usually a high pointed P-wave (P-pulmonale) along with right-axis deviation of QRS and a modified T-wave reflecting right ventricular overload [63]. The CAT scan shows an elevated heart apex due to the right ventricular dilatation. The high pointed P-waves imply right atrial enlargement and are generally associated with pulmonary hypertension. Pulmonary hypertension is the hallmark reaction in HAPE [58] and hence the focus of oxygen treatment of HAPE. Blood circulation is shunted away from poorly oxygenated lung zones towards healthy alveoli to minimize V/Q mismatch upon exposure to high altitude hypoxia [64]. Echocardiography can show a dilated right ventricle and right atrium, as well as increased pulmonary artery pressure [65]. Chest X-ray and/or CAT scans can often show a convex pulmonary artery in the P-A projection (Figure 7). In the city of La Paz, Bolivia, at 3100–4100 m, the Mean Pulmonary Artery Pressure (MPAP) is 23 mmHg [66]. Hence, pulmonary hypertension is a normal physiologic favorable response at high altitude in over 200 million inhabitants. Peñaloza et al. affirmed, “Pulmonary hypertension and right ventricular hypertrophy are not associated with any kind of symptoms in high-altitude children, and we must understand these changes as related in some way to the mechanisms of natural acclimatization” [67].

## 15. Benefits of Life under Chronic Hypoxia

Chronic hypoxia is not deleterious but rather beneficial under many circumstances. It gives us significant advantages: extended longevity [9], less mortality from several diseases [68,69], paradoxically greater tolerance to hypoxia as one goes higher [43], a form of human plasticity with lower incidence of cardiac diseases [70], less obesity, less asthma [9,71], fewer hypertension problems in the Andeans [72,73], less lung cancer incidence [74,75], and even less COVID-19 incidence and lower Case Fatality Rate, as we had first correctly predicted before the arrival of the pandemic to Bolivia, later also confirmed by others [25,76,77,78]. This was also observed in the questions above, because there is misconception among some of the sea level high altitude experts, (due of their biased understanding), that life at high altitude is always “deleterious”. Chronic hypoxia preconditioning has also been beneficial in rodent studies in protecting myocardial infarction [78], vascular remodeling, and strokes [79]. We believe sea-level dwellers have a severe survival disability: poor tolerance to hypoxia. Whereas, we, the highlanders, have achieved the first giant step, through physiologic adaptation to chronic hypoxia, of what we have termed BioSpaceForming (adaptation to life in space) [21]. Humanity is preparing to colonize Mars. We foretell that humans in space will become a permanent chronic hypoxia species, which is why we have proposed that space travel should be carried out in a chronic hypoxia environment [21,22].

## 16. Conclusions

High-altitude illnesses are best evaluated by physicians working on site, gaining experience over many years resulting in favorable recoveries as those expressed in this paper. High-altitude parameters of normality are different from those at sea level. High-altitude biology follows physics laws, which apply to the distention of gases, the partial pressures of oxygen and carbon dioxide, and the acid-base balance. The treatment of high-altitude illness becomes easier when there is a profound knowledge of high-altitude physiology and the Oxygen Transport Triad. The high-altitude pulmonary hypertension within normal high-altitude parameters is a normal response aiding in oxygen exchange at high altitude. Oxygen administration in HAPE reduces pulmonary artery pressure and recovery ensues.

## Figures and Tables

**Table 1:** Calculated arterial oxygen content (CaO_2_) differences between sea level and high altitude. PB = barometric pressure, SaO_2_ = arterial hemoglobin oxygen saturation, Hb = hemoglobin, PaO_2_= arterial oxygen partial pressure, Hb O_2_% is the fraction of oxygen transported by hemoglobin, and dissolved O_2_ is the fraction of O_2_ dissolved in plasma.

	PB mmHg	SaO_2_ %	Hb gm/dL	PaO_2_ mmHg	CaO_2_ Vol%	Hb O_2_ %	Dissolved O_2_ %
**Sea-level**	760	99	15	100	20	98.45	1.55
**La Paz (3500 m)**	495	90	17	60	20.50	99.13	0.87
